# Development and validation of deep learning- and ensemble learning-based biological ages in the NHANES study

**DOI:** 10.3389/fnagi.2025.1532884

**Published:** 2025-07-16

**Authors:** Yushu Huang, Xifan Yang, Qi Wang, Adila Abula, Yue Dong, Wenyuan Li

**Affiliations:** ^1^Center of Clinical Big Data and Analytics of The Second Affiliated Hospital, Department of Big Data in Health Science School of Public Health, Zhejiang University School of Medicine, Hangzhou, Zhejiang, China; ^2^Zhejiang Provincial Key Laboratory of Intelligent Preventive Medicine, Hangzhou, Zhejiang, China

**Keywords:** aging, biological age, machine learning, deep learning, deep neural networks

## Abstract

**Introduction:**

Conventional machine learning (ML) approaches for constructing biological age (BA) have predominantly relied on blood-based markers, limiting their scope. This study aims to develop and validate novel ML-based BA models using a comprehensive set of clinical, behavioral, and socioeconomic factors and evaluate their predictive performance for mortality.

**Methods:**

We analyzed data from 24,985 participants in the National Health and Nutrition Examination Survey (NHANES) from 1999 to 2010, with follow-up extending to 31 December 2019, or until death or loss to follow-up. Thirty features, including blood and urine biochemistry, physical examination data, behavioral traits, and socioeconomic factors, were selected using the Least Absolute Shrinkage and Selection Operator (LASSO). These features were utilized to train deep neural networks (DNN) and ensemble learning models, specifically the Deep Biological Age (DBA) and Ensemble Biological Age (EnBA), with chronological age (CA) as the reference label. Model performance was assessed using mean absolute error (MAE), while interpretability was explored using Shapley Additive exPlanation (SHAP). Predictive accuracy of DBA and EnBA for mortality was compared with Phenotypic Age (PhenoAge) using the area under the curve (AUC) derived from Cox proportional hazards models and hazard ratios (HR), adjusted for demographics and lifestyle factors. Sensitivity analyses were performed to ensure robustness.

**Results:**

DBA and EnBA accurately predicted actual age (MAE = 2.98 and 3.58 years, respectively) and demonstrated strong predictive capability for all-cause mortality, with AUCs of 0.896 (95% CI: 0.891–0.898) for DBA and 0.889 (95% CI: 0.884–0.894) for EnBA. Higher DBA and EnBA accelerations were significantly associated with increased mortality risk (HR = 1.059 and 1.039, respectively). SHAP analysis highlighted prescription medication usage, hepatitis B surface antibody status, and vigorous physical activity as the most influential features contributing to DBA predictions. Furthermore, BA acceleration was linked to elevated risk of death from specific chronic conditions, including cardiovascular and cerebrovascular diseases and cancer.

**Discussion:**

Our study successfully developed and validated two ML-based BA models capable of accurately predicting both all-cause and cause-specific mortality. These findings suggest that the DBA and EnBA models hold promise for early identification of high-risk individuals, potentially facilitating timely preventive interventions and improving population health outcomes.

## 1 Introduction

Aging is a complicated and inevitable process, which is closely associated with functional limitations, chronic diseases (Jiang et al., 2024), and disabilities (Christensen et al., 2009). The degree of aging shows significant individual differences, so human beings of the same Chronological Age (CA) may have varied physiological aging (Partridge et al., 2018). This difference has sparked growing interest in accurately estimating aging (Moqri et al., 2023). Biological age (BA), compared to CA or other aging assessment models, is constructed based on multiple biomarkers (Moqri et al., 2023; Moqri et al., 2024; Putin et al., 2016; Xue et al., 2021). Therefore, BA more accurately reflects an individual’s aging process. Biological age acceleration (Age-Acc), derived as the residual from regressing BA on CA, quantifies deviations from expected aging trajectories, where positive values indicate accelerated aging (BA exceeding CA) and negative values reflect decelerated aging. Accurate estimation is crucial for better understanding factors that may influence the aging process, laying the foundation for improving individual healthspan and the development of geroprotectors and identifying populations at higher risk of mortality (López-Otín et al., 2023).

The selection and inclusion range of biomarkers are always critical factors limiting the precision of BA estimation (Moqri et al., 2023). Previous studies mainly used blood biochemistry indicators (Mamoshina et al., 2019; Putin et al., 2016) and DNAm data (Sathyan et al., 2023) as sources of biomarkers for constructing aging clocks, such as the classic aging methods the Klemera-Doubal Method (KDM) (Klemera and Doubal, 2006; Machado et al., 2024) and Phenotypic Age (PhenoAge) (Gao et al., 2023; Jia et al., 2024; Levine et al., 2018; Mak et al., 2023; Roberts et al., 2021; Wang et al., 2020; Wang et al., 2023). However, blood biochemistry indicators can only reflect an individual’s health status at a specific moment and are easily affected by short-term factors (Duan et al., 2022). While methylation aging indicators delivered the most precise CA estimation currently available, epigenetic information outside of early life is too stable to quantify the influences of behaviors, living conditions, environment, and therapeutic approaches on biological aging rates (Murabito et al., 2018; Shipony et al., 2014). Nevertheless, both behavioral and socioeconomic factors play significant roles in influencing the aging process (Argentieri et al., 2025; Huang et al., 2025; Lawrence et al., 2020). The exploration of multidimensional aging assessment models, which comprehensively assess blood biochemistry indicators, urine biochemistry indicators, physical examination data, behavioral information, and demographic and socio-economic characteristics, remains limited.

In the past, the estimation of BA primarily relied on multiple linear regression (MLR) and principal component analysis (PCA) methods, which were constrained by dimensionality disasters and the complex correlation structure of indicators. Machine learning (ML) and deep learning (DL) are capable of uncovering complex patterns and non-linear relationships in large datasets (Dong et al., 2021). DNN leverages hierarchical feature interactions and automatically handles heterogeneous data types, enabling the discovery of complex synergistic effects that are difficult to capture with traditional methods (Huang et al., 2025). Additionally, Ensemble Learning approach enhances robustness by combining diverse base models, mitigating overfitting risks while leveraging their complementary strengths (Zhao et al., 2023). Over the past decade, they have proven to be powerful tools for constructing BA (Bobrov et al., 2018; Galkin et al., 2021; Gialluisi et al., 2022; Mamoshina et al., 2018; Mamoshina et al., 2019; Putin et al., 2016). These studies, however, are often limited by singularity of data dimensions (Galkin et al., 2021; Mamoshina et al., 2018; Mamoshina et al., 2019; Putin et al., 2016), a lack of interpretability (Mamoshina et al., 2019), and a lack of systematic hyperparameter tuning (Gialluisi et al., 2022). These shortcomings not only lead to potential vast deviations in the identification of key biomarkers but also to deficiencies in prediction accuracy [MAE = 5.55 (Putin et al., 2016), 5.94 (Mamoshina et al., 2018), 6.00 (Gialluisi et al., 2022) years]. Moreover, the complex internal structure of ML, while facilitating the modeling of non-linear relationships, also implies a reduction in the interpretability of the predictions. SHAP, grounded in the Shapley values from game theory, allocates the extent of all of feature’s contribution to the model’s predictions through considering all possible feature combinations (Lundberg and Lee, 2017). It addressed feature interactions and provided a more effective solution to the black-box problem of ML (Tseng et al., 2020; Xue et al., 2021).

Our study first developed a novel Deep Biological Age (DBA) model and Ensemble Biological Age (EnBA), and validated them by linking them to death risk among 24,985 participants from the NHANES database, monitored over roughly 20 years. Biological aging was determined based on blood and urine biochemistry indicators, physical examination data, behavioral information, and demographic and socio-economic characteristics obtained at baseline.

## 2 Materials and methods

### 2.1 Study population

The dataset used in this research came from NHANES, alongside the basic information and methods described earlier. All NHANES participants completed health questionnaires, physical examinations, and laboratory tests on blood and urine samples.

Based on prior experience, we utilized aggregated data from the 1999 to 2010 NHANES survey cycles, which included a comprehensive analysis of biomarkers and covariates. After deleting people under 20 years of age and those with missing data on disease status, the remaining data were processed to assess other missingness. For continuous variables, multiple imputation was applied, while categorical variables were imputed using the expectation-maximization (EM) algorithm. Following these procedures, the final analysis encompassed 24,985 participants. We then developed two measures of biological aging: DBA and EnBA, using complete datasets from the selected biomarkers and covariates.

Among the 24,985 participants, individuals diagnosed with any of eight pathological conditions (diabetes, cardiac insufficiency, coronary artery disease (CHD), chest pain, myocardial necrosis, stroke, cancer or malignancies, chronic kidney disease) were grouped as patient cohort, while the rest formed the healthy cohort (*N* = 15,011). We assumed that in the healthy cohort, BA is close to CA, and therefore used this cohort to train the DNN and Ensemble models. The trained models were then applied to the entire population to estimate individual BA. Flowchart of the sample selection were showed in Supplementary Figure 1.

Participants in the cohort were all required to give their written informed consent.

### 2.2 Covariate data collection

Demographic data (including age, gender, personal earnings, ethnic background, level of education, and marital status), health behavior (related to sleep, exercise routine, tobacco use, and drinking habits), and personal and family health histories (notably of cardiovascular diseases, cancer, and diabetes) were systematically gathered through comprehensive questionnaires by trained professionals. Physical examinations were conducted using precision-calibrated instruments according to established protocols to measure Body Mass Index (BMI), fat mass percentage, systolic and diastolic pressure, and beats per minute (BPM). Standardized procedures were employed to obtain fasting blood and urine samples for subsequent laboratory analyses.

Variables such as BMI (classifications as < 25, 25–30, and ≥ 30 kg/m^2^), ethnicity (classified as Mexican-origin American, Hispanic of other origins, Non-Hispanic White, and Non-Hispanic Black), poverty-to-income ratio (categorized as < 1 or ≥ 1), education level (classified as elementary school education or below, secondary school education or below, and college education or higher), marital status (distinguished between unmarried or other and married or cohabiting), smoking status (classified as never, former, or current), alcohol consumption patterns (classified as yes or no), sleep status (classified as trouble or not), and physical activity levels (categorized as moderate work, vigorous work, or none) were incorporated as covariates in the analysis. Specially, Physical activity levels were derived from self-reported participation, following definitions from questions provided by the NHANES: (1) Over the past 30 days, did you/SP do moderate activities for at least 10 min that cause only light sweating or a slight to moderate increase in breathing or heart rate? Some examples are brisk walking, bicycling for pleasure, golf, and dancing. (2) Over the past 30 days, did you/SP do any vigorous activities for at least 10 min that caused heavy sweating, or large increases in breathing or heart rate? Some examples are running, lap swimming, aerobics classes or fast bicycling.

### 2.3 Mortality ascertainment

Mortality data, including causes of death such as cancer, chronic lower respiratory conditions, diabetes mellitus, Alzheimer’s disease, cardiovascular and cerebrovascular diseases, influenza and pneumonia, nephritis, nephrotic syndrome, and nephrosis, were obtained from NHANES follow-up records. These data were available through the Public-use Linked Mortality Files (LMF) for NHANES.

The research timeframe commenced on the enrollment date, terminating upon the first occurrence of mortality, dropout, withdrawal from the study, or upon reaching the study cutoff date of 31 December 2019. The details of the subsequent analytical procedures have been described in earlier publications (Huang et al., 2023; Zhang et al., 2021). Briefly, participant health data were linked to mortality outcomes and specific causes up to 31 December 2019, using unique identifiers assigned to participants. Mortality outcomes and follow-up periods, expressed in years, were provided for all participants.

### 2.4 Deep Biological Age (DBA)

Based on previous research and LASSO selection, thirty features were selected for training (Supplementary Table 1). The data from the healthy cohort was split into training (*N* = 12,009) and testing (*N* = 3,002) datasets with an 8:2 ratio. A DNN model (learning rate = 0.1, hidden layer sizes: 700, 1000, 700, 200, and 1) was applied, using actual age as the dependent variable, with 10-fold cross-validation. The network incorporated dropout regularization (rate = 0.4) to mitigate overfitting, as detailed in Supplementary Table 2. To isolate biometric signals, we further developed a biometric-only DBA model by excluding five behavioral and socioeconomic features (e.g., personal income, physical activity, alcohol level, country of birth and education level), retaining identical hyperparameters as the full-feature model. MAE was computed to evaluate the predictive accuracy of the model. The SHAP method was used to evaluate each feature’s contribution to DBA and to determine the variable importance ranking.

### 2.5 Ensemble Biological Age (EnBA)

We also trained ensemble learning models using data from the healthy cohort following a similar approach. The data were split into training and testing datasets with an 8:2 ratio. The ensemble models were constructed using a combination of base learners, including Random Forest, Extra Trees, XGBoost, and Support Vector Machine (SVM). We selected Ridge regression as the meta learner due to its ability to perform well in scenarios where multicollinearity is present and its capacity to prevent overfitting through L2 regularization. Ridge regression’s regularization parameter, set to α = 0.1, controls the model’s complexity, helping to maintain a balance between bias and variance, as detailed in Supplementary Table 2. To validate the contribution of non-biometric traits, we additionally implemented a biometric-only EnBA model, excluding the same five behavioral/socioeconomic features while retaining the full-feature model’s hyperparameters.

The ensemble model’s effectiveness was assessed using MAE (10-fold cross-validation) to assess their accuracy in predicting actual age and to determine the differences between the predicted BA and CA.

### 2.6 PhenoAge estimation

As previously mentioned, PhenoAge V2 was developed by regressing the mortality risk on 42 blood biomarkers and CA and has been widely used to capture morbidity and mortality risks across different subgroups. PhenoAge (V2) was computed using the R package “BioAge” (Kwon and Belsky, 2021). Based on the Gompertz distribution, a parametric proportional hazards model was established by selecting chronological age and nine clinical biomarkers, converting the 10 years mortality risk into years of age. Initially, we trained the algorithm on the NHANES III dataset, modeling mortality as a function of 12 blood biomarkers [including albumin (ALB), alkaline phosphatase (ALP), blood urea nitrogen (BUN), creatinine (CRE), C-reactive protein (CRP), HbA1c, total cholesterol (TC), uric acid (UA), white blood cell count (WBC), lymphocyte percentage (LYM%), mean cell volume (MCV), and red cell distribution width (RDW)], and finally projected the biological aging measures in our data.

Additionally, we reconstructed the PhenoAge V1 developed by Levine el al. (2018) to enable direct comparison with earlier-generation clocks after excluding participants without blood glucose (*n* = 12,925). This version combines CA and nine core biomarkers [albumin (ALB), alkaline phosphatase (ALP), creatinine (CRE), C-reactive protein (CRP), blood glucose (BG), white blood cell count (WBC), lymphocyte percentage (LYM%), mean cell volume (MCV), and red cell distribution width (RDW)], reflecting systemic physiological dysregulation as originally described (Levine et al., 2018). Both V1 and V2 were evaluated alongside our DBA and EnBA clocks (Supplementary Figure 2), highlighting performance differences between survival-optimized clocks (PhenoAge) and age-trained models (DBA/EnBA).

### 2.7 Biological age acceleration

To calculate biological age acceleration (Age-Acc), we performed linear regression of each biological age measure (DBA, EnBA, and PhenoAge) against chronological age (CA), with the resulting residuals representing age acceleration values (DBA-Acc, EnBA-Acc, and PhenoAge-Acc, respectively); positive values indicate accelerated aging (biological age exceeding expected age) while negative values reflect decelerated aging, with all models incorporating necessary adjustments for technical covariates.

### 2.8 Statistical analysis

Study participant’ baseline characteristics were summarized using median (IQR) or N (%), and statistical tests were employed to detect differences: *t*-tests and Wilcoxon rank-sum tests for continuous variables, as appropriate; chi-square tests for categorical variables.

We compared the predictive performance of DBA, EnBA, PhenoAge (V1 and V2) with all-cause mortality using Cox models and used AUC to evaluate performance. Sensitivity analyses were undertaken to ascertain the robustness of these associations, with further refinements made to the models by adjusting for additional factors: actual age, gender, level of education, BMI, sleep status, physical activity levels, smoking behavior, and alcohol consumption.

To determine the associations between Age and Age-Acc, and both all-cause and cause-specific mortality, we employed Cox proportional hazards models, making comparable adjustments in each. To further solidify these associations, we conducted multiple sensitivity analyses. Finally, we presented the association analysis between DBA Acceleration and cause-specific mortality using a forest plot.

All analytical tasks and visualizations were carried out utilizing Python version 3.11.5 and R version 4.5.3. The *P*-values were calculated on a two-tailed basis, and statistical significance was established at *p* < 0.05.

## 3 Results

### 3.1 Characteristics of study population

The fundamental attributes of the study participants at baseline are outlined in Table 1. This investigation included 24,985 individuals, with 15,011 participants designated as healthy, representing the entirety of this cohort. The median age of the healthy cohort was 38, while the median age of all participants was 46 years. The gender distribution was balanced, with females comprising 52.8% of the healthy cohort and 52.4% of the all participants.

**TABLE 1:** Characteristics of study population.

Characteristics	Healthy (*n*/%)	All (*n*/%)
Total	15,011	24,985
Age [median (IQR)]	38 [28.0.50.0]	46 (33.0, 62.0)
**Gender (%)**		
Male	7,078 (47.2)	11,900 (47.6)
Female	7,933 (52.8)	13,085 (52.4)
**Ethnicity level (%)**		
Mexican American	3,484 (23.2)	5,074 (20.3)
Non-Hispanic White	7,133 (47.5)	12,479 (49.9)
Other Hispanic	2,619 (17.4)	4,762 (19.1)
Non-Hispanic Black	1,775 (11.8)	2,670 (10.7)
**Family PIR (%)**		
< 1	3,131 (20.9)	5,040 (20.2)
≥ 1	11,880 (79.1)	19,945 (79.8)
**Education level (%)**		
Primary school degree or less	3,979 (26.5)	7,216 (28.9)
High school degree or less	3,478 (23.2)	5,881 (23.6)
College degree or higher	7,539 (50.3)	11,862 (47.5)
**Marital status (%)**		
Unmarried or other	5,448 (36.9)	9,181 (36.7)
Married or living with a partner	9,563 (63.7)	15,804 (63.3)
**Smoking (%)**		
Current	2,942 (19.6)	5,519 (22.1)
Former	3,668 (24.4)	6,247 (25.0)
Never	8,396 (56.0)	13,210 (52.9)
**Alcohol (%)**		
Yes	4,021 (26.8)	7,429 (29.7)
No	10,990 (73.2)	17,556 (70.3)
**Trouble sleeping (%)**		
Yes	1,556 (10.4)	3,396 (13.6)
No	13,455 (89.6)	21,589 (86.4)
**Physical activity (%)**		
Moderate work	3,820 (25.4)	6,570 (26.3)
None	6,586 (43.9)	11,980 (47.9)
Vigorous work	4,605 (30.7)	6,435 (25.8)
**BMI (kg/m^2^, %)**		
< 25	5,424 (36.1)	7,529 (30.1)
≥ 30	5,392 (35.9)	8,660 (34.7)
25–30	4,195 (27.9)	8,796 (35.2)
**Death and causes**		
All-cause (%)	978 (6.5)	4,032 (16.1)
Cardiovascular and cerebrovascular death (%)	367 (2.4)	1,773 (7.1)
Cancer (%)	375 (2.5)	1,239 (5.0)
Diabetes (%)	23 (0.2)	209 (0.8)
Chronic lower respiratory diseases (%)	95 (0.6)	331 (1.3)
Alzheimer’s disease (%)	72 (0.5)	230 (0.9)
Influenza and pneumonia (%)	25 (0.2)	124 (0.5)
Nephritis nephrotic syndrome and nephrosis (%)	21 (0.1)	126 (0.5)

The median DBA was 45.35 (IQR: 30.18; 95% CI: 21.58, 79.63), demonstrating a strong correlation with chronological age (R^2^ = 0.95, *p* < 0.0001). The median EnBA was 45.71 (IQR: 30.30; 95% CI: 22.32, 81.76), showing a strong correlation with chronological age (R^2^ = 0.93, *p* < 0.0001). The median PhenoAge V1 was 35.99 (IQR: 29.43; 95% CI: 37.71, 38.19), indicating a strong correlation with chronological age (R^2^ = 0.91, *p* < 0.0001). The median PhenoAge V2 was 43.29 (IQR: 30.40; 95% CI: 16.07, 82.56), indicating a strong correlation with chronological age (R^2^ = 0.92, *p* < 0.0001). The median DBA-Acc was −0.289, with an IQR of 2.41. The median EnBA-Acc was −0.139, with an IQR of 5.36. The median PhenoAge-Acc V2 was −0.917, with an IQR of 6.31. The median PhenoAge-Acc V2 was −0.254, with an IQR of 6.24.

### 3.2 Prediction performance of biological age

**FIGURE 1:**
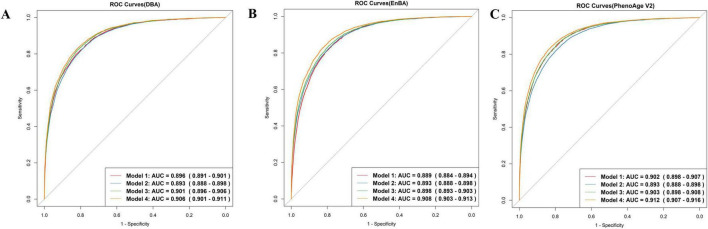
ROC curves. Model 1 predicts mortality based on Deep Biological Age (DBA) **(A)**, Ensemble Biological Age (EnBA) **(B)**, Phenotypic Age Version 2 (PhenoAge V2) **(C)**. Model 2 is based on chronological age (CA). Model 3 is based on Model 1, adjusting for CA and gender. Model 4 is based on Model 3, further adjusting for education, BMI, drinking, smoking, sleep, and physical activity.

Both DBA and EnBA successfully forecasted CA, with DBA attaining an MAE of 2.98 years and EnBA achieving an MAE of 3.58 years. After excluding five characteristics, the biometric-only versions of DBA and EnBA achieved MAEs of 3.26 and 4.59 years, respectively. Furthermore, DBA and CA demonstrated comparable performance in predicting mortality, with DBA achieving an AUC of 0.896 (95% CI: 0.891–0.901) and CA achieving an AUC of 0.893 (95% CI: 0.888–0.898) (Figure 1A, Models 1 and 2). When gender and CA were added to the model based on DBA (Figure 1A, Model 3), the AUC slightly increased to 0.901 (95% CI: 0.896–0.906). Model 4, which included DBA, CA, gender, education level, BMI, alcohol use, smoking status, physical activity, and sleep status, showed an AUC of 0.906 (95% CI: 0.901–0.911).

Ensemble Biological Age and PhenoAge V2 were also evaluated using Cox proportional hazards models (Figures 1B, C). Both demonstrated strong predictive performance for mortality prediction. The AUC for EnBA was 0.889 (95% CI: 0.884–0.894), while the AUC for PhenoAge V2 was 0.902 (95% CI: 0.898–0.907). Furthermore, Age-Acc alone did not demonstrate strong predictive power for mortality, as shown by DBA-Acc with an AUC of 0.530 (95% CI: 0.520–0.541) and EnBA-Acc with an AUC of 0.549 (95% CI: 0.538–0.560). However, after adjusting for the same covariates as the Age-based model, the Age-Acc-based model showed similarly excellent predictive performance, with DBA-Acc achieving an AUC of 0.908 (95% CI: 0.903–0.912).

These findings illustrated that while BAs are a significant predictor of mortality, the integration of additional sociodemographic variables substantially improved the predictive accuracy of the regression model. The improvement in performance due to these covariate adjustments was particularly pronounced in the Age-Acc-based models. Furthermore, we selected the top 10 variables based on SHAP values and included them as predictors in the Cox proportional hazards model. The resulting ROC curve achieved an AUC of 0.81 (95% CI: 0.802–0.817). The biometric-only DBA model achieved an AUC of 0.892 (95% CI: 0.885–0.9) in predicting all-cause mortality, whereas its full-feature counterpart demonstrated significantly improved discriminative ability [AUC = 0.902 (95% CI: 0.895–0.91)] (Supplementary Figure 2). Similarly, the full-feature EnBA model outperformed its biometric-only version [AUC = 0.895 (0.887–0.902) vs. 0.884 (0.876–0.891)], highlighting the added value of integrating non-biometric domains—such as income level and alcohol use—into aging clocks. These results align with the observed performance gap between full-feature and biometric-only models, where the inclusion of behavioral and socioeconomic traits enhanced predictive accuracy by ∼1% (ΔAUC = 0.01). Notably, the full-feature DBA model exhibited superior AUC compared to PhenoAge V1 [AUC = 0.901 (0.894–0.908)], underscoring its potential as a more robust biomarker of aging-related mortality risk.

### 3.3 Association with all-cause mortality

Adopting the same approach, we explored the associations between Age-Acc and mortality rates (Table 2). In our full cohort (*n* = 24,985, 4,032 deaths), after accounting for various covariates including age, gender, education level, BMI, alcohol consumption, smoking, sleep, and physical activity, the link between Age-Acc and mortality risk remained statistically significant. Each additional year of DBA-Acc was associated with a 5.9% increase in mortality risk (HR = 1.059, 95% CI: 1.050–1.068). Similarly, EnBA-Acc correlated with a 3.9% rise in mortality risk (HR = 1.039, 95% CI: 1.032–1.045), while PhenoAge-Acc V2 showed a 7.3% augmentation in mortality risk (HR = 1.073, 95% CI: 1.067–1.079).

**TABLE 2:** Associations of age acceleration (Age-Acc) with all-cause mortality.

Age-Acc	Participants	Number of death	HR (95% CI)
DBA-Acc	24,985	4,032	1.059 (1.050, 1.068)
EnBA-Acc	24,985	4,032	1.039 (1.032, 1.045)
PhenoAge-Acc V2	24,985	4,032	1.073 (1.067, 1.079)
DBA-Acc	12,060	1,979	1.066 (1.052, 1.080)
EnBA-Acc	12,060	1,979	1.036 (1.027, 1.046)
DBA (biometric only)-Acc	12,060	1,979	1.032 (1.025, 1.038)
EnBA (biometric only)-Acc	12,060	1,979	1.026 (1.017, 1.035)
PhenoAge-Acc V2	12,060	1,979	1.073 (1.067, 1.079)
PhenoAge-Acc V1	12,060	1,979	1.055 (1.049, 1.061)

HR, hazard ratio; CI, confidence interval. The model was based on age accelerations, adjusting for age, gender, education level, BMI, smoking, drinking, sleep, and exercise status.

In the sub cohort without blood glucose (*n* = 12,060), biometric-only models demonstrated reduced predictive power compared to full-feature versions: DBA (Biometric Only)-Acc had a minimal HR of 1.032 (95% CI: 1.025–1.038), whereas full DBA-Acc retained a higher HR of 1.066. Similarly, EnBA (Biometric Only)-Acc (HR = 1.026) underperformed relative to EnBA-Acc (HR = 1.036).

Additional analysis of models’ evolution revealed distinct patterns across adjustment models. In Model 1 (unadjusted), DBA demonstrated the highest hazard ratio (HR = 1.123) among all metrics, outperforming PhenoAge V1 (HR = 1.084) and PhenoAge V2 (HR = 1.103). After adjusting for chronological age and gender (Model 2), DBA retained superior predictive power (HR = 1.066) compared to PhenoAge V1 (HR = 1.056). Further multivariable adjustment (Model 3, including education, BMI, lifestyle factors) marginally reduced HRs for most metrics, but DBA still showed the strongest association (HR = 1.053) among biomarker-based clocks, significantly surpassing PhenoAge V1 (HR = 1.044).

Notably, the Biometric Only versions of DBA and EnBA exhibited stark performance declines. For instance, DBA (Biometric Only) showed a near-null HR in Model 3 (HR = 0.998, 95% CI: 0.998–0.999), indicating loss of predictive validity after adjusting for non-biometric confounders.

In contrast, the full-feature DBA and EnBA models maintained robust associations (DBA: HR = 1.053; EnBA: HR = 1.036), underscoring the critical role of behavioral and socioeconomic domains in refining risk estimates. Similarly, EnBA (Biometric Only) demonstrated weaker HRs across all models (e.g., Model 3: HR = 1.039) compared to its full-feature counterpart (HR = 1.036), though the gap was smaller than in DBA. These findings confirm that integrating biometric baselines with contextual factors significantly enhances the predictive validity of biological age clocks, with DBA emerging as the top-performing metric against PhenoAge V1.

### 3.4 Cause-specific mortality

**FIGURE 2:**
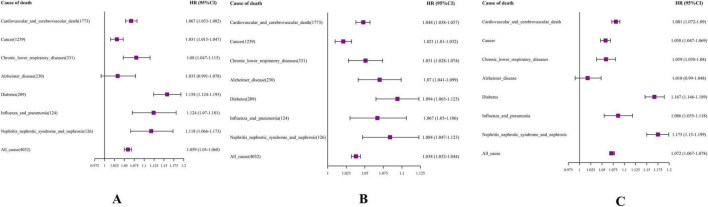
Forest plot. **(A)** DBA-Acc; **(B)** EnBA-Acc; **(C)** PhenoAge-Acc V2. The model was based on age acceleration (Age-Acc), adjusting for age, gender, education level, e Body Mass Index (BMI), smoking, drinking, sleep, and exercise status.

Utilizing Cox proportional hazards models, we uncovered significant correlations between DBA-Acc and heightened cause-specific mortality rates from cardiovascular and cerebrovascular diseases, cancer, diabetes, chronic lower respiratory diseases, influenza and pneumonia, nephritis, nephrotic syndrome, and nephrosis (Figure 2A).

More precisely, an increment of one year in DBA-Acc notably elevated the risk of mortality stemming from cardiovascular and cerebrovascular diseases (HR = 1.067, 95% CI: 1.053–1.082). DBA-Acc was also significantly associated with an increased mortality rate for individuals dying from cancer, with the HR rising by 3.1% (HR = 1.031, 95% CI: 1.015–1.047).

Ensemble Biological Age-Acc showed statistically significant associations with the mortality risk of all specific chronic diseases monitored in the NHANES dataset (Figure 2B). The results for PhenoAge Acceleration V2 were similar to those for DBA-Acc, showing significant associations with increased mortality risk from various chronic diseases, except for Alzheimer’s disease (Figure 2C).

### 3.5 SHAP for DBA

**FIGURE 3:**
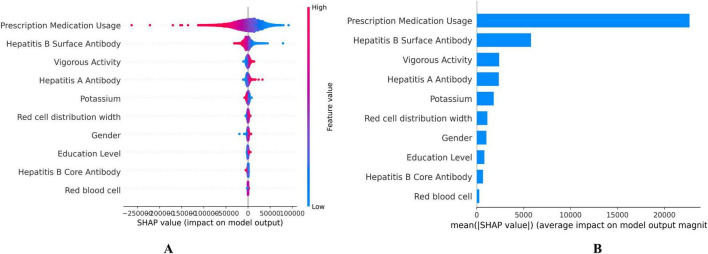
Shapley Additive exPlanation (SHAP) values on the DNNs model. **(A)** SHAP summary plot, **(B)** Feature importance ranking by mean SHAP value. Each dot represents a single participant’s SHAP value (impact on predicted biological age). Color indicates feature value (red = high, blue = low). Position on the x-axis shows whether that value contributes to increasing (positive, right) or decreasing (negative, left) biological age.

The SHAP values for each feature’s contribution to DBA were illustrated in Figure 3, showing the top 10 most significant variables selected by the DBA model (Figure 3A). These values quantified the impact of each variable on the cumulative predictive performance, with higher values indicating greater influence. Each dot represents a single participant’s SHAP value (impact on predicted BA); Color indicates feature value (red = high, blue = low); Position on the x-axis shows whether that value contributes to increasing (positive, right) or decreasing (negative, left) BA. Consistent with expectations, the key biomarkers reflecting medical conditions and prescription medication usage emerged as the primary predictor among all evaluated features, followed by hepatitis B surface antibody, vigorous activity, and hepatitis A antibody. Prescription medication use was assessed in NHANES via a binary question covering all therapeutic classes except vitamins and minerals. Elevated serum creatinine levels were linked to a heightened risk of mortality, underscoring that potential liver function impairment is a major factor contributing to decreased DBA (Figure 3B).

## 4 Discussion

In this study, we developed and validated two novel aging clocks, the DBA model and EnBA, using DNN and ensemble learning techniques, leveraging data from NHANES, including blood and urine biochemical indicators, physical examination data, behavioral information, and demographic and socio-economic characteristics. Our findings demonstrated that both DBA and EnBA alone exhibit outstanding predictive performance in mortality prediction. This comprehensive approach not only deepened our understanding of the factors influencing aging but also provided supports to interventions aimed at promoting healthy aging and reducing premature mortality.

Identifying aging biomarkers is crucial for assessing an individual’s physiological aging, with the fundamental concept tracing back to the first biological age proposition in 1988, extending to contemporary research (Baker and Sprott, 1988). Our study considered comprehensive feature inclusion approaches and selected 30 features, including biochemistry, behaviors and socioeconomic agents which impact aging trajectories and mortality risk (Argentieri et al., 2025; Huang et al., 2025; Lawrence et al., 2020). Previous studies have often focused on identifying key aging factors in blood biomarkers or other laboratory tests for constructing aging clocks (Mamoshina et al., 2018; Mamoshina et al., 2019; Putin et al., 2016). For instance, Mamoshina constructed an aging clock utilizing blood test data from populations in three different regions, with external validation through NHANES using Cox regression in all-cause mortality risk [MAE = 9.93, 95% CI: (10.36–9.35)] (Mamoshina et al., 2018). Over the years, PhenoAge was innovatively developed by screening biomarkers associated with mortality, directly related to diseases or mortality rather than relying on CA predictions (Jia et al., 2024; Wang et al., 2016; Wang et al., 2023). This method, often referred to as the Levine Method, utilizes a set of clinical biomarkers along with CA to estimate biological age. Moreover, while the Levine Method considers CA and clinical biomarkers, the calculation of PhenoAge requires follow-up and mortality data from the study population (Vetter et al., 2022). Compared to PhenoAge V1 and V2, our DBA and EnBA, which consider a broader range of indicators, more accurately predict changes in actual age (Pearson’s r 0.97 vs. 0.96 and 0.96, R^2^ 0.95 vs. 0.92 and 0.93), and do not rely on follow-up data, offering greater clinical applicability.

In the past, researchers have developed biological age models using methods ranging from initial multiple linear regression (Cabral et al., 2022) and PCA (Nakamura et al., 1988) to more complex approaches like the Levine method (Jia et al., 2024; Wang et al., 2023). These methods were limited by the complex non-linear relationships in large datasets and struggle to maintain close correlation with CA while providing robust mortality prediction capabilities. ML and DL algorithms excelled in autonomously deriving models and identifying complex patterns from extensive datasets, proving superior to traditional methods through their powerful predictive abilities (Mudabbiruddin and Mosavi, 2023). Gialluisi et al. (2022) initially developed a DNN-based aging clock using 33 circulating blood biomarkers in an Italian population, tested for predicting all-cause mortality [MAE = 6.47; HR = 1.05, 95% CI: (1.04–1.05)]. We developed the DBA model, which exhibited excellent performance in the association of its acceleration with all-cause mortality risk [HR = 1.059, 95% CI: (1.050–1.068)], following adjustments for various covariates such as CA, gender, education level, BMI, alcohol consumption, smoking, sleep, and physical activity. In terms of predicting mortality after adjusting for baseline covariates, the DBA model achieved an AUC of 0.902, slightly lower than that of PhenoAge V2 (AUC = 0.906) but superior to EnBA (AUC = 0.895) and PhenoAge V1 (AUC = 0.901). Our study conducted long-term follow-up in a general population and expanded the range of included features, making DBA more accurate than previous similar studies (MAE = 2.98). Notably, the Biometric Only versions of DBA (AUC = 0.892) and EnBA (AUC = 0.884) exhibited stark performance declines. At the same time, we linked DBA-Acc with cause-specific mortality risk.

Our study identified the top 10 features intrinsically linked to aging through the SHAP method, with Prescription Medication Usage, Hepatitis B Surface Antibody, Vigorous Activity ranking as the top three contributors (Figure 3). When the top 10 features were used as variables in the Cox regression model, the corresponding ROC curve had an AUC of 0.802 (95% CI: 0.792–0.813). It is widely accepted that improved healthcare conditions lead to significant increases in lifespan and reductions in mortality (Nayan et al., 2018). Hepatitis B Surface Antibody (HBsAb) indicates the degree of Hepatitis B Virus (HBV) infection, serving as a pivotal factor in the morbidity and mortality linked to chronic hepatitis B (CHB), which ranks among the top ten causes of death worldwide (Huang et al., 2023; Iloeje et al., 2007). Approximately 15%–25% of individuals who are chronic carriers of HBV develop chronic liver disease, which may include cirrhosis, liver failure, or liver cancer, which are the primary pathways through which HBV infection impacts aging and mortality (Ganem and Prince, 2004). A UK Biobank (UKB) study using wearable devices and machine learning found that brief, intense non-exercise physical activity was significantly associated with a 38%–40% decrease in all-cause and cancer mortality risk, as well as a 48%–49% reduction in cardiovascular disease mortality risk (Stamatakis et al., 2022). Research has also shown that around one hour of vigorous activity per week is the optimal dose for reducing health risks (Ahmadi et al., 2022).

Our study encompassed a broad cohort of 24,985 individuals, spanning various age demographics, each with comprehensive clinical biomarker data. A methodological advantage was the application of the SHAP method, which addresses the challenge of interpretability constraints in opaque algorithmic models (Ribeiro et al., 2016). Rigorous analytical methods were implemented, including ten-fold cross-validation on the training set and internal validation on the test cohort. Compared to the Levine Method, which requires follow-up and mortality data for the calculation of PhenoAge, our DBA and EnBA models offer a significant advantage by relying solely on cross-sectional data and over performed to original PhenoAge version. Notably, clinicians can easily access the data for the key factors identified in our model through electronic health records, eliminating the need for additional documentation or supplementary diagnostic procedures for patients.

Despite its strengths, this study acknowledges certain limitations. First, although the evaluation of BAs encompasses multiple dimensions, the NHANES dataset has a limited scope of data collection, precluding a comprehensive assessment that includes various modalities such as diagnostic imaging. This limitation may affect the model’s accuracy and precision. Moreover, since this study was conducted within the United States population, the fitting and extrapolation potential of this predictive model to other racial and regional populations remain unverified.

## 5 Conclusion

Our study developed and validated two biological age estimators using blood and urine biomarkers, physical exam data, behavioral information, and socio-economic characteristics. These estimators accurately predicted all-cause and specific chronic disease mortality among NHANES participants and were compared with PhenoAge. Notably, our models have shown better performance than original PhenoAge version at predicting mortality. Although slightly less effective at predicting mortality than PhenoAge V2, our models aligned more closely with actual age and did not require follow-up data, making them practical for use with physical exams and electronic health records. Further research is needed to validate these models across different populations and optimize them with multimodal data for a more comprehensive assessment of aging.

## Data Availability

The datasets presented in this study can be found in online repositories. The names of the repository/repositories and accession number(s) can be found below: https://wwwn.cdc.gov/nchs/nhanes/continuousnhanes/default.aspx?BeginYear=1999.

## References

[B1] Ahmadi M. Clare P. Katzmarzyk P. Del Pozo Cruz B. Lee I. M. Stamatakis E. (2022). Vigorous physical activity, incident heart disease, and cancer: How little is enough? *Eur. Heart J.* 43 4801–4814. 10.1093/eurheartj/ehac572 36302460 PMC9726449

[B2] Argentieri M. Amin N. Nevado-Holgado A. Sproviero W. Collister J. Keestra S. (2025). Integrating the environmental and genetic architectures of aging and mortality. *Nat. Med.* 31 1016–1025. 10.1038/s41591-024-03483-9 39972219 PMC11922759

[B3] Baker G. Sprott R. (1988). Biomarkers of aging. *Exp. Gerontol.* 23 223–239. 10.1016/0531-5565(88)90025-3 3058488

[B4] Bobrov E. Georgievskaya A. Kiselev K. Sevastopolsky A. Zhavoronkov A. Gurov S. (2018). PhotoAgeClock: Deep learning algorithms for development of non-invasive visual biomarkers of aging. *Aging* 10 3249–3259. 10.18632/aging.101629 30414596 PMC6286834

[B5] Cabral D. Bigliassi M. Cattaneo G. Rundek T. Pascual-Leone A. Cahalin L. (2022). Exploring the interplay between mechanisms of neuroplasticity and cardiovascular health in aging adults: A multiple linear regression analysis study. *Auton Neurosci.* 242:103023. 10.1016/j.autneu.2022.103023 36087362 PMC11012134

[B6] Christensen K. Doblhammer G. Rau R. Vaupel J. (2009). Ageing populations: The challenges ahead. *Lancet* 374 1196–1208. 10.1016/S0140-6736(09)61460-4 19801098 PMC2810516

[B7] Dong S. Wang P. Abbas K. (2021). A survey on deep learning and its applications. *Comput. Sci. Rev.* 40:100379. 10.1016/j.cosrev.2021.100379

[B8] Duan R. Fu Q. Sun Y. Li Q. (2022). Epigenetic clock: A promising biomarker and practical tool in aging. *Ageing Res. Rev.* 81:101743. 10.1016/j.arr.2022.101743 36206857

[B9] Galkin F. Mamoshina P. Kochetov K. Sidorenko D. Zhavoronkov A. (2021). DeepMAge: A methylation aging clock developed with deep learning. *Aging Dis.* 12 1252–1262. 10.14336/AD.2020.1202 34341706 PMC8279523

[B10] Ganem D. Prince A. (2004). Hepatitis B virus infection–natural history and clinical consequences. *N. Engl. J. Med.* 350 1118–1129. 10.1056/NEJMra031087 15014185

[B11] Gao X. Geng T. Jiang M. Huang N. Zheng Y. Belsky D. (2023). Accelerated biological aging and risk of depression and anxiety: Evidence from 424,299 UK Biobank participants. *Nat. Commun.* 14:2277. 10.1038/s41467-023-38013-7 37080981 PMC10119095

[B12] Gialluisi A. Di Castelnuovo A. Costanzo S. Bonaccio M. Persichillo M. Magnacca S. (2022). Exploring domains, clinical implications and environmental associations of a deep learning marker of biological ageing. *Eur. J. Epidemiol.* 37 35–48. 10.1007/s10654-021-00797-7 34453631

[B13] Huang S. Li X. Chen C. Ning Q. Huang J. (2023). Effect of Anti-HBs on mortality among resolved HBV infection: A population-based prospective cohort study. *Infect. Dis. Ther.* 12 871–890. 10.1007/s40121-023-00766-5 36754951 PMC10017907

[B14] Huang Y. Da L. Dong Y. Li Z. Liu Y. Li Z. (2025). Construction and validation of a DNN-based biological age and its influencing factors in the China Kadoorie Biobank. *Geroscience* 47 4241–4252. 10.1007/s11357-025-01577-x 40050560 PMC12181170

[B15] Iloeje U. Yang H. Jen C. Su J. Wang L. You S. (2007). Risk and predictors of mortality associated with chronic hepatitis B infection. *Clin. Gastroenterol. Hepatol.* 5 921–931. 10.1016/j.cgh.2007.06.015 17678844

[B16] Jia Q. Chen C. Xu A. Wang S. He X. Shen G. (2024). A biological age model based on physical examination data to predict mortality in a Chinese population. *iScience* 27:108891. 10.1016/j.isci.2024.108891 38384842 PMC10879664

[B17] Jiang M. Tian S. Liu S. Wang Y. Guo X. Huang T. (2024). Accelerated biological aging elevates the risk of cardiometabolic multimorbidity and mortality. *Nat. Cardiovasc. Res.* 3 332–342. 10.1038/s44161-024-00438-8 39196113 PMC13265155

[B18] Klemera P. Doubal S. (2006). A new approach to the concept and computation of biological age. *Mech. Ageing Dev.* 127 240–248. 10.1016/j.mad.2005.10.004 16318865

[B19] Kwon D. Belsky D. W. (2021). A toolkit for quantification of biological age from blood chemistry and organ function test data: Bioage. *Geroscience* 43 2795–2808. 10.1007/s11357-021-00480-5 34725754 PMC8602613

[B20] Lawrence K. Kresovich J. O’Brien K. Hoang T. Xu Z. Taylor J. (2020). Association of neighborhood deprivation with epigenetic aging using 4 clock metrics. *JAMA Netw. Open* 3:e2024329. 10.1001/jamanetworkopen.2020.24329 33146735 PMC7643028

[B21] Levine M. Lu A. Quach A. Chen B. Assimes T. Bandinelli S. (2018). An epigenetic biomarker of aging for lifespan and healthspan. *Aging* 10 573–591. 10.18632/aging.101414 29676998 PMC5940111

[B22] López-Otín C. Blasco M. Partridge L. Serrano M. Kroemer G. (2023). Hallmarks of aging: An expanding universe. *Cell* 186 243–278. 10.1016/j.cell.2022.11.001 36599349

[B23] Lundberg S. Lee S. I. (2017). A unified approach to interpreting model predictions. *arXiv [Preprint]* 10.48550/arXiv.1705.07874

[B24] Machado A. Silva J. Colosimo E. Needham B. Maluf C. Giatti L. (2024). Clinical biomarker-based biological age predicts deaths in Brazilian adults: The ELSA-Brasil study. *Geroscience* 46 6115–6126. 10.1007/s11357-024-01186-0 38753229 PMC11494676

[B25] Mak J. McMurran C. Kuja-Halkola R. Hall P. Czene K. Jylhävä J. (2023). Clinical biomarker-based biological aging and risk of cancer in the UK Biobank. *Br. J. Cancer* 129 94–103. 10.1038/s41416-023-02288-w 37120669 PMC10307789

[B26] Mamoshina P. Kochetov K. Cortese F. Kovalchuk A. Aliper A. Putin E. (2019). Blood biochemistry analysis to detect smoking status and quantify accelerated aging in smokers. *Sci. Rep.* 9:142. 10.1038/s41598-018-35704-w 30644411 PMC6333803

[B27] Mamoshina P. Kochetov K. Putin E. Cortese F. Aliper A. Lee W. (2018). Population specific biomarkers of human aging: A big data study using South Korean, Canadian, and Eastern European patient populations. *J. Gerontol. A Biol. Sci. Med. Sci.* 73 1482–1490. 10.1093/gerona/gly005 29340580 PMC6175034

[B28] Moqri M. Herzog C. Poganik J. Justice J. Belsky D. (2023). Biomarkers of aging for the identification and evaluation of longevity interventions. *Cell* 186 3758–3775. 10.1016/j.cell.2023.08.003 37657418 PMC11088934

[B29] Moqri M. Herzog C. Poganik J. Ying K. Justice J. Belsky D. (2024). Validation of biomarkers of aging. *Nat. Med.* 30 360–372. 10.1038/s41591-023-02784-9 38355974 PMC11090477

[B30] Mudabbiruddin M. Mosavi A. (2023). “Machine learning and mathematical models for prediction of structural aging process,” in *Proceedings of the 2023 IEEE 17th International Symposium on Applied Computational Intelligence and Informatics (SACI)*, (Piscataway, NJ: IEEE).

[B31] Murabito J. Zhao Q. Larson M. Rong J. Lin H. Benjamin E. (2018). Measures of biologic age in a community sample predict mortality and age-related disease: The framingham offspring study. *J. Gerontol. A Biol. Sci. Med. Sci.* 73 757–762. 10.1093/gerona/glx144 28977464 PMC5946832

[B32] Nakamura E. Miyao K. Ozeki T. (1988). Assessment of biological age by principal component analysis. *Mech. Ageing Dev.* 46 1–18. 10.1016/0047-6374(88)90109-1 3226152

[B33] Nayan M. Juurlink D. Austin P. Macdonald E. Finelli A. Kulkarni G. (2018). Medication use and kidney cancer survival: A population-based study. *Int. J. Cancer* 142 1776–1785. 10.1002/ijc.31204 29226327

[B34] Partridge L. Deelen J. Slagboom P. (2018). Facing up to the global challenges of ageing. *Nature* 561 45–56. 10.1038/s41586-018-0457-8 30185958

[B35] Putin E. Mamoshina P. Aliper A. Korzinkin M. Moskalev A. Kolosov A. (2016). Deep biomarkers of human aging: Application of deep neural networks to biomarker development. *Aging* 8 1021–1033. 10.18632/aging.100968 27191382 PMC4931851

[B36] Ribeiro M. T. Singh S. Guestrin C. (2016). ““Why should I trust you?”: Explaining the predictions of any classifier,” in *Proceedings of the 22nd ACM SIGKDD International Conference on Knowledge Discovery and Data Mining*, (San Francisco, CA: Association for Computing Machinery), 1135–1144.

[B37] Roberts J. Vittinghoff E. Lu A. Alonso A. Wang B. Sitlani C. (2021). Epigenetic age and the risk of incident atrial fibrillation. *Circulation* 144 1899–1911. 10.1161/CIRCULATIONAHA.121.056456 34587750 PMC8671333

[B38] Sathyan S. Ayers E. Adhikari D. Gao T. Milman S. Barzilai N. (2023). Biological age acceleration and motoric cognitive risk syndrome. *Ann. Neurol.* 93 1187–1197. 10.1002/ana.26624 36843279 PMC10865507

[B39] Shipony Z. Mukamel Z. Cohen N. Landan G. Chomsky E. Zeliger S. (2014). Dynamic and static maintenance of epigenetic memory in pluripotent and somatic cells. *Nature* 513 115–119. 10.1038/nature13458 25043040

[B40] Stamatakis E. Ahmadi M. Gill J. Thøgersen-Ntoumani C. Gibala M. Doherty A. (2022). Association of wearable device-measured vigorous intermittent lifestyle physical activity with mortality. *Nat. Med.* 28 2521–2529. 10.1038/s41591-022-02100-x 36482104 PMC9800274

[B41] Tseng P. Chen Y. Wang C. Chiu K. Peng Y. Hsu S. (2020). Prediction of the development of acute kidney injury following cardiac surgery by machine learning. *Crit. Care* 24:478. 10.1186/s13054-020-03179-9 32736589 PMC7395374

[B42] Vetter V. Drewelies J. Sommerer Y. Kalies C. Regitz-Zagrosek V. Bertram L. (2022). Epigenetic aging and perceived psychological stress in old age. *Transl. Psychiatry* 12:410. 10.1038/s41398-022-02181-9 36163242 PMC9513097

[B43] Wang C. Koutrakis P. Gao X. Baccarelli A. Schwartz J. (2020). Associations of annual ambient PM2.5 components with DNAm PhenoAge acceleration in elderly men: The normative aging study. *Environ. Pollut.* 258:113690. 10.1016/j.envpol.2019.113690 31818625 PMC7044052

[B44] Wang G. Han C. Detrick B. Casolaro V. Levine D. Fried L. (2016). Herpesvirus infections and risk of frailty and mortality in older women: Women’s health and aging studies. *J. Am. Geriatr. Soc.* 64 998–1005. 10.1111/jgs.14090 27131018 PMC4882224

[B45] Wang S. Wen C. Li W. Li S. Sun M. Xu A. (2023). Development of a novel multidimensional measure of aging to predict mortality and morbidity in the prospective MJ Cohort. *J. Gerontol. A Biol. Sci. Med. Sci.* 78 690–697. 10.1093/gerona/glac161 35921680

[B46] Xue B. Li D. Lu C. King C. Wildes T. Avidan M. (2021). Use of machine learning to develop and evaluate models using preoperative and intraoperative data to identify risks of postoperative complications. *JAMA Netw. Open* 4:e212240. 10.1001/jamanetworkopen.2021.2240 33783520 PMC8010590

[B47] Zhang Y. Chen C. Pan X. Guo J. Li Y. Franco O. (2021). Associations of healthy lifestyle and socioeconomic status with mortality and incident cardiovascular disease: Two prospective cohort studies. *BMJ* 373:n604. 10.1136/bmj.n604 33853828 PMC8044922

[B48] Zhao C. Wu D. Huang J. Yuan Y. Zhang H. Peng R. (2023). BoostTree and boostforest for ensemble learning. *IEEE Trans. Pattern. Anal. Mach. Intell.* 45 8110–8126. 10.1109/TPAMI.2022.3227370 37015516

