# Supplementary material for: Development and validation of deep learning- and ensemble learning-based biological ages in the NHANES study
Source: Front Aging Neurosci. 2025 Jul 16;17:1532884. doi: 10.3389/fnagi.2025.1532884 (PMC12307447; doi:10.3389/fnagi.2025.1532884)
Supplement: Supplementary file 1 [file Table_1.docx]

Supplementary Material

**Table S1.** LASSO-Selected Features and Their Coefficients in the Model

| Features | LASSO coefficient | |
| --- | --- | --- |
| Estimated Glomerular Filtration Rate | | 10.699689 |
| Serum creatinine | | 4.225698 |
| Systolic BP | | 2.758049 |
| Gender | | 1.905343 |
| Personal Income | | 1.692892 |
| Prescription Medication Usage | | 1.665196 |
| Hepatitis A Antibody | | 1.488055 |
| Blood lead | | 1.356283 |
| Glycohemoglobin Percent | | 1.242994 |
| Urinary creatinine | | 1.191784 |
| Height | | 1.1557 |
| Red cell distribution width | | 1.124844 |
| Mean cell volume | | 1.094325 |
| Hepatitis B Surface Antibody | | 1.068777 |
| Vigorous activity | | 1.065192 |
| Alcohol Level | | 1.043831 |
| Serum osmolality | | 0.88765 |
| Serum uric acid | | 0.869968 |
| Hepatitis B Core Antibody | | 0.862569 |
| Red blood cell count | | 0.847443 |
| Serum phosphorus | | 0.789413 |
| Bicarbonate | | 0.759329 |
| Serum chloride | | 0.758872 |
| Total cholesterol | | 0.747823 |
| Platelet count | | 0.733572 |
| Potassium | | 0.701334 |
| Albumin | | 0.700057 |
| Country of birth | | 0.645903 |
| Pulse Regular/Irregular | | 0.634768 |
| Education Level | | 0.549479 |

**Table S2. Optimized Hyperparameters for Deep and Ensemble Learning** **Models**

| Model Type | Hyperparameter | Best Value |
| --- | --- | --- |
| DNN | Learning Rate | 0.1 |
|  | Hidden Layer Sizes | 700, 1000, 700, 200 |
|  | Dropout Rate | 0.4 |
|  | Loss Function | MAE |
|  | Cross-Validation | Ten-fold |
| Ensemble Learning |  |  |
|  | Random Forest |  |
|  | max depth | 11 |
|  | max leaf nodes | 70 |
|  | min samples leaf | 8 |
|  | Extra Trees |  |
|  | max depth | 11 |
|  | max leaf nodes | 65 |
|  | min samples leaf | 8 |
|  | XGBoost |  |
|  | learning rate | 0.07 |
|  | min child weight | 4 |
|  | subsample | 0.9 |
|  | SVM |  |
|  | C | 74 |
|  | gamma | 1.00E-05 |
|  | CatBoost |  |
|  | l2 leaf reg | 7 |
|  | iterations | 100 |


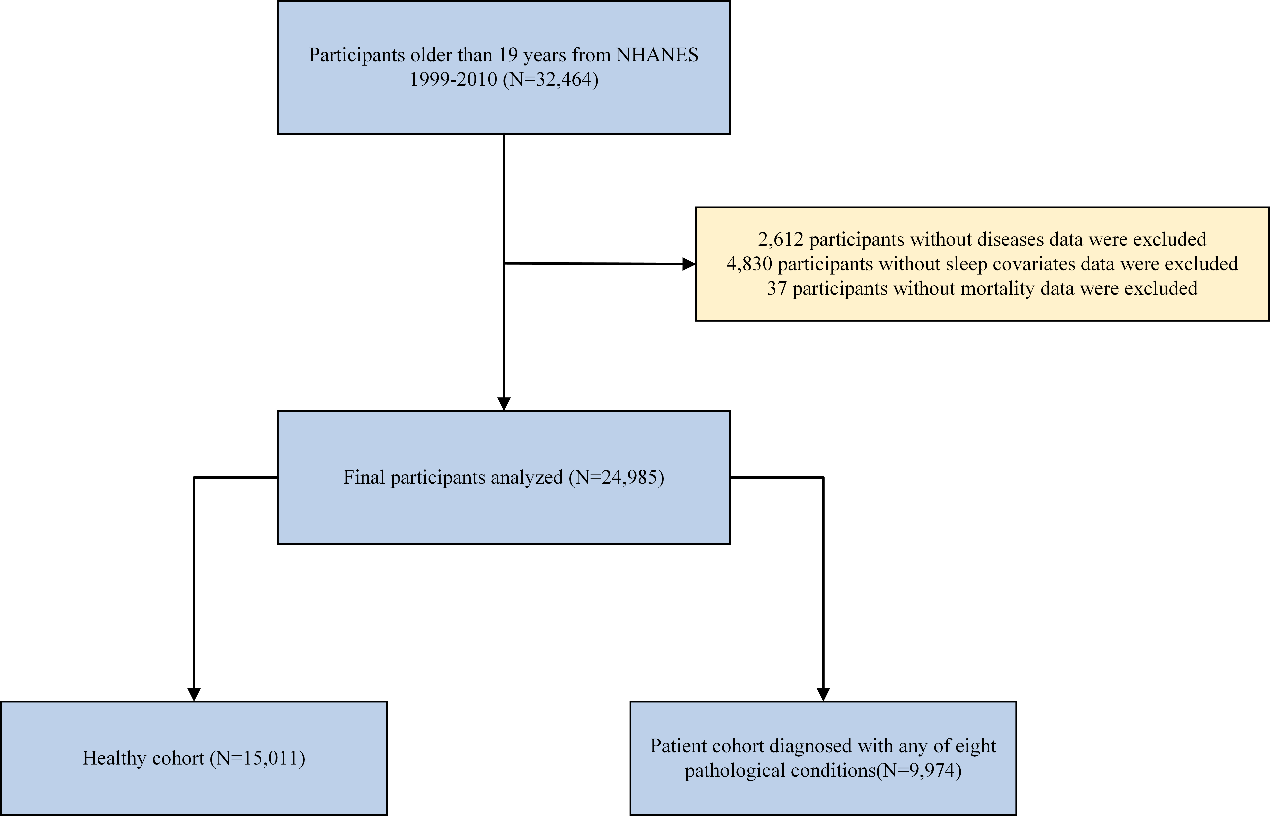


**Figure S1.** Flowchart of the sample selection.


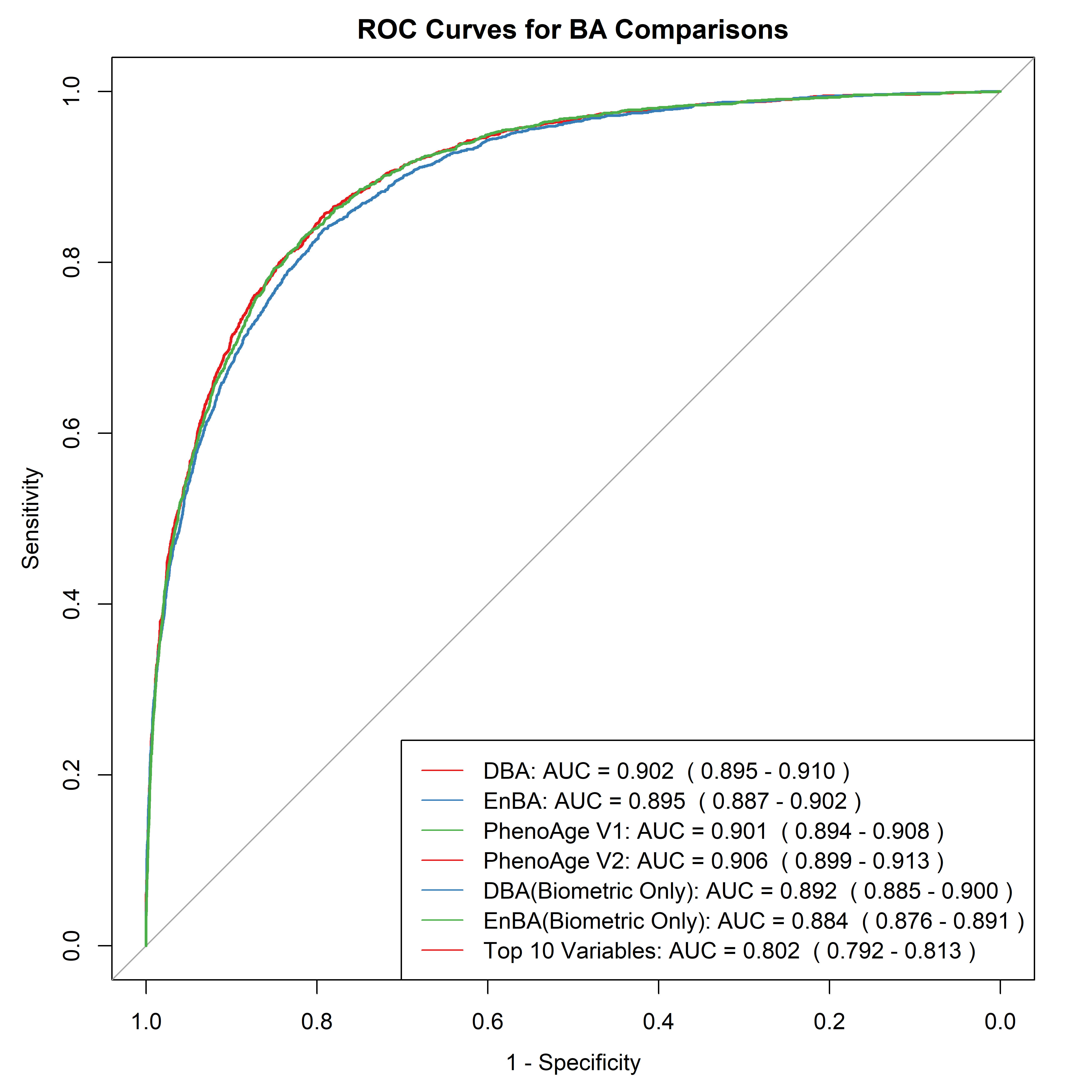


**Figure S2.** ROC Curves for Comparisons, among DBA, EnBA, PhenoAge, and top 10 variables.
